# Non-linear RR interval metrics in canine atrial fibrillation and their relationship to 24-h mean heart rate

**DOI:** 10.3389/fcvm.2026.1890025

**Published:** 2026-07-16

**Authors:** Tamilselvam Gunasekaran, Robert A. Sanders, Nicholas B. Olivier

**Affiliations:** Department of Small Animal Clinical Sciences, College of Veterinary Medicine, Michigan State University, East Lansing, MI, United States

**Keywords:** atrial fibrillation, canine, Holter monitoring, Jensen's inequality, rate-control threshold, RR interval irregularity

## Abstract

**Introduction:**

In both dogs and humans with atrial fibrillation (AF), rate control is monitored using the 24-h Holter mean heart rate (meanHR), which reflects only the central tendency of the RR interval distribution. Naturally occurring canine AF provides a translational model because of its comparable AV nodal physiology and autonomic regulation.

**Methods:**

Fifty client-owned dogs with confirmed AF underwent 24-h Holter monitoring. Ten non-linear RR metrics were evaluated: coefficient of variation of RR (CVRR), Poincaré SD1, SD2, SD1/SD2 ratio, |ΔRR|, turning-point ratio, DFA-α, sample entropy, and multiscale entropy (MScEn). The Jensen-gap index (*D*) was also calculated; rate dependence was assessed using Spearman correlation and the within-quintile IQR ratio.

**Results and Discussion:**

The metrics were partitioned into rate-coupled (CVRR, SD1, SD2, |ΔRR|, DFA-α; |*ρ*| = 0.64–0.91, *R*^2^ = 0.42–0.75) and rate-independent (SD1/SD2 ratio, turning-point ratio, MScEn; |*ρ*| < 0.14, *R*^2^ < 0.01) classes. All 50 dogs showed a negative Jensen gap (D: mean −13.4 bpm, range −29.9 to −5.8; *ρ* with CVRR = −0.934), with no discontinuity observed at 125 bpm. Five dogs were reclassified as non-rate-controlled based on medianHR. Within the rate-controlled group, CVRR ranged from 0.285 to 0.748. Rate-independent metrics differed between pre- and postdrug dogs (SD1/SD2 *p* = 0.020; MScEn *p* = 0.032). The 125-bpm threshold used to assess rate control in dogs dichotomizes a continuous relationship and collapses heterogeneous RR distributions, limitations that have direct parallels in human AF rate-control monitoring. Future studies in both canine and human AF should incorporate rate-independent metrics alongside meanHR and specify the HR computation method.

## Introduction

Atrial fibrillation (AF) is the most common sustained cardiac arrhythmia in both humans and dogs, and ventricular rate control is the principal therapeutic strategy for most patients in both species. Clinical monitoring of rate control relies on a single 24-h Holter-derived mean heart rate (meanHR), a scalar measure that captures only the average ventricular response and does not characterize the structure or complexity of the underlying beat-to-beat variation in ventricular rhythm. In human AF, landmark trials, including RACE II, have established that heart rate reduction improves outcomes. However, debate persists regarding the optimal rate target and whether the mean rate alone adequately characterizes treatment response (1).

Dogs with naturally occurring AF, arising in the context of dilated cardiomyopathy (DCM), myxomatous mitral valve disease, or in large and giant breeds with structurally normal hearts (lone AF), provide a uniquely valuable translational model (2). Unlike induced or anesthesia-based models, naturally occurring canine AF is chronic, spontaneous, and autonomically intact and managed using the same 24-h Holter monitoring technology used in human clinical practice. The Optimal Rate Control in Dogs with AF (ORCA) study established that a meanHR ≤125 bpm is associated with significantly prolonged survival, and this threshold has become the clinical target for rate control in dogs (3). A follow-up study confirmed that drug titration to achieve this target is associated with favorable cardiac remodeling (4). This canine rate-control threshold directly parallels the human rate target (RACE II: lenient resting heart rate <110 bpm), positioning canine AF as a relevant platform for evaluating rate-control monitoring in both species.

However, meanHR = 60,000/mean(RR) captures only the central tendency of the RR interval distribution. It does not describe whether RR intervals cluster, exhibit long-range correlations, vary in complexity across timescales, or follow symmetric distributions. These distributional properties, quantified by non-linear RR metrics, may reflect AV nodal physiology and autonomic modulation independently of heart rate. A drug that reduces meanHR could produce identical heart rate values through fundamentally different effects on the structure of RR irregularities. Furthermore, meanHR is not uniquely defined: because the reciprocal function (1/*x*) is convex, the arithmetic mean of beat-by-beat instantaneous heart rates systematically exceeds 60,000/mean(RR) by an amount proportional to RR dispersion, a discrepancy quantified by Jensen's inequality (5). When Holter software reports the former, the computed HR may differ by clinically meaningful amounts, potentially altering rate-control classification in both dogs and humans.

This study had three objectives: first, to determine whether commonly reported non-linear RR interval metrics remain independent of heart rate across the clinical range of meanHR in dogs with AF; second, to evaluate the Jensen-gap index (*D*) as a measure of RR irregularity and to assess whether the 125-bpm threshold constitutes a biologically meaningful boundary with direct implications for rate-control monitoring in both canine and human AF; and third, to determine whether rate-control drugs alter the structure of RR irregularity beyond their heart rate-lowering effect using predrug vs. postdrug comparisons.

## Materials and methods

### Study population

Medical records of dogs diagnosed with AF that underwent Holter monitoring between 2010 and 2026 were retrospectively reviewed. The diagnosis of AF was established by in-clinic or referral electrocardiography demonstrating the absence of organized *P* waves and an irregularly irregular ventricular rhythm. All dogs underwent 24-h ambulatory Holter monitoring at the Cardiology Service, Department of Small Animal Clinical Sciences, Michigan State University College of Veterinary Medicine. Recordings were acquired using a Lifecard CF digital three- or four-electrode Holter monitor (Spacelabs Healthcare, Redmond, WA, USA) and analyzed using commercial software (Pathfinder, Spacelabs Healthcare) by a board-certified veterinary cardiologist. Beat-by-beat RR intervals and ventricular premature complex (VPC) annotations were exported for secondary analysis. The exported data consisted of tabular beat-series files containing beat-to-beat RR intervals (ms) and beat-type annotations, which were processed in R for all secondary computations. All metrics were computed from the full annotated beat series, inclusive of VPCs. VPC burden was expressed as V% (the proportion of VPC beats per recording). All included dogs had persistent or permanent AF confirmed by Holter monitoring. Holter recordings were classified as predrug (no rate-control medication at the time of Holter evaluation) or postdrug (receiving at least one rate-control drug at the time of Holter evaluation) based on contemporaneous medical records and further subclassified according to clinical designation (routine/asymptomatic evaluation vs. uncontrolled, where Holter was performed when the dog was experiencing clinical symptoms), yielding four groups.

### Conventional HR descriptors

MeanHR was computed as 60,000/mean(RR). MedianHR was computed as the median of 60,000/RRᵢ across all beats. The coefficient of variation of RR (CVRR) was calculated as SD(RR)/mean(RR). The median and interquartile range (IQR) of |ΔRR| = |RR(i) − RR(i − 1)| were computed. Minimum and maximum 1-minute meanHRs were derived by binning beats into consecutive 1-min intervals by cumulative RR time, retaining minutes with ≥10 beats.

### Non-linear RR metrics

Poincaré SD1 and SD2 were computed according to established definitions: SD1 = √(0.5 × SD[ΔRR]) and SD2 = √(2 × SD[RR]^2^ − 0.5 × SD[ΔRR]^2^) (6). The SD1/SD2 ratio was used as a scale-independent measure of Poincaré plot ellipticity. The turning-point ratio was used as a simple non-linear measure of RR irregularity. A turning point was defined at index *i* if (RR[*i*] − RR[*i* − 1]) × (RR[*i*] − RR[*i* + 1]) > 0, that is, a beat whose interval was either strictly greater than or strictly less than both its immediate neighbors. The turning-point ratio was computed as the number of such turning points divided by (*n* − 2), where *n* denotes the total number of beats in the RR series. Computationally intensive metrics, including sample entropy (SampEn), multiscale entropy (MScEn), and detrended fluctuation analysis (DFA), used non-overlapping 5,000-beat windows; windows containing <3,000 beats were excluded; dog-level values were defined as the median across all eligible windows. SampEn was computed using an embedding dimension of *m* = 2 and a tolerance of *r* = 0.2 ×  SD (7), with the analysis capped at 2,000 points per window. MScEn was calculated across scales 1–10 using coarse-graining (non-overlapping block means), with SampEn calculated at each scale; the MScEn coefficient was defined as the slope of SampEn vs. log(scale) and required ≥3 valid scales (8, 9). DFA-α was estimated using first-order detrending over logarithmically spaced scales [16 to min (1,024, *n*/4) beats] and was calculated as the slope of log *F*(*s*) vs. log(*s*) (10). All computations were performed in R using the data.table, readxl, zoo, and non-linearTseries packages.

### Jensen-gap index (*D*)

For each dog, *X* = 60,000/mean(RR) (standard Holter meanHR), whereas *Y* = mean (60,000/RRᵢ) (mean instantaneous HR). Jensen's inequality for convex *f*(*x*) = 1/*x* guarantees E[1/RR] ≥ 1/E[RR], implying that *Y* ≥ *X* and *D* = *X* − *Y* ≤ 0 whenever RR varies (5). A second-order Taylor expansion yields *D*/*X* ≈ −CVRR^2^, linking *D* directly to RR dispersion. The overestimation *E* = −*D* (bpm) quantifies how much a Holter system overestimates meanHR by averaging individual RR values rather than computing HR from the mean RR.

### Rate-dependence classification

Rate dependence of each metric was quantified using (1) Spearman’s *ρ* with continuous meanHR; (2) *R*^2^ from simple linear regression of the metric on meanHR; and (3) the within-HR-quintile IQR ratio, in which dogs were grouped into meanHR quintiles and the mean within-bin IQR was expressed as a fraction of the overall IQR. A ratio approaching 1.0 indicates that nearly all metric variability persists after controlling for HR, whereas a low ratio indicates that the metric is largely determined by heart rate. Metrics were classified as statistically significant, meaningfully rate-coupled (statistically significant *R*^2^ ≥ 0.25), or rate-independent (non-significant *R*^2^).

### Statistical analysis

Normality was assessed using the Shapiro–Wilk test. Continuous data are reported as median [IQR] for non-normally distributed variables and mean ± SD for approximately normal variables. Spearman correlation coefficients between all metric pairs were computed to assess inter-metric collinearity. Rate-coupled metrics were highly intercorrelated (|*ρ*| = 0.85–0.97 among SD1, SD2, and |ΔRR|), making multivariable modeling inadvisable. Clinical subgroup comparisons, including predrug vs. postdrug and rate-controlled (RC; meanHR ≤ 125 bpm) vs. not rate-controlled (NRC), were performed using two-sided Mann–Whitney *U*-tests (*α* = 0.05). A partial Spearman correlation between Jensen's index (*D*) and rate-control status was computed after adjusting for continuous meanHR by linear regression. All analyses were exploratory, and no correction for multiple comparisons was applied.

## Results

### Study population

Fifty dogs were included in the final population (Table 1). The cohort consisted predominantly of large and giant breeds, including Great Danes (*n* = 9, 18%), mixed-breed dogs (*n* = 6, 12%), Golden Retrievers (*n* = 5, 10%), and Bullmastiffs (*n* = 4, 8%). Underlying cardiac disease was present in 48/50 (96%) dogs, including DCM in 23 (46%), myxomatous mitral valve disease (DMVD) in 12 (24%), equivocal echocardiographic findings to either classify as DCM or DMVD in 9 (18%), subaortic stenosis in 2 (4%), patent ductus arteriosus in 2 (4%), and lone AF in 2 (4%). A history of left-sided congestive heart failure (CHF) was documented in 22/50 (44%) dogs and right-sided heart failure in 7/50 (14%). However, this does not indicate that Holter recordings were performed when the dogs were in active CHF and only reflects their clinical history. Twenty-seven dogs were receiving antiarrhythmic drugs, with the following rate control drugs: diltiazem monotherapy (*n* = 16), combination therapy with diltiazem and digoxin (*n* = 10), sotalol monotherapy (*n* = 3), diltiazem plus atenolol (*n* = 2), and combination therapy with diltiazem, digoxin, and atenolol (*n* = 1).

**Table 1:** Study population characteristics and Holter-derived metrics (*n* = 50 dogs).

Variable	Median [IQR]	Range	Mean ± SD
Signalment			
Age (years)	6.5 [4.5–9.2]	2.0–14.1	6.9 ± 3.0
Body weight (kg)	45.0 [30.0–65.4]	15.0–79.4	47.7 ± 20.0
Sex: neutered male/female/intact male	31/13/6	—	—
Holter context			
Predrug Holter, *n* (%)	23 (46%)	—	—
Rate-controlled (meanHR ≤125 bpm), *n* (%)	19 (38%)	—	—
VPC burden >5%, *n* (%)	18 (36%)	—	—
Holter metrics			
MeanHR (bpm)	129.7 [116.8–156.0]	60.8–206.3	133.3 ± 33.3
MedianHR (bpm)	140.5 [123.8–161.3]	64.4–215.8	144.1 ± 32.4
MeanHR vs. medianHR discrepancy (bpm)	—	—	10.7 ± 8.9
MeanRR (ms)	462.6 [384.5–513.8]	290.9–986.3	482.9 ± 142.9
Jensen gap *D* (bpm)	−11.2 [−16.7, −8.1]	−29.9 to −5.8	−13.4 ± 6.4
Non-linear RR metrics			
CVRR	0.309 [0.247–0.442]	0.194–0.710	0.348 ± 0.127
SD1 (ms)	115.1 [78.4–192.6]	55.0–420.6	149.7 ± 96.7
SD2 (ms)	167.8 [105.3–243.6]	72.7–632.5	207.4 ± 137.5
SD1/SD2 ratio	0.728 [0.667–0.802]	0.548–0.929	0.734 ± 0.085
Median |ΔRR| (ms)	77.0 [56.0–100.3]	42.0–302.0	89.2 ± 49.4
IQR |ΔRR| (ms)	110.0 [81.3–175.5]	63.0–576.0	159.9 ± 122.0
Turning-point ratio	0.644 [0.635–0.656]	0.518–0.695	0.643 ± 0.028
DFA-α	0.697 [0.648–0.782]	0.521–0.932	0.714 ± 0.106
Sample entropy	1.881 [1.685–1.974]	1.154–2.091	1.809 ± 0.224
MScEn coefficient	0.001 [−0.038–0.038]	−0.131–0.160	−0.001 ± 0.063

VPC, ventricular premature complex; CVRR, coefficient of variation of RR; SD1/SD2, Poincaré plot axes; |ΔRR|, absolute successive RR difference; DFA-α, detrended fluctuation analysis exponent; MScEn, multiscale entropy. Jensen gap *D*=HR from meanRR − mean_instantaneous_HR; all values *D* < 0 (Jensen's inequality).

### Rate-dependence classification of non-linear metrics

**Figure 1:**
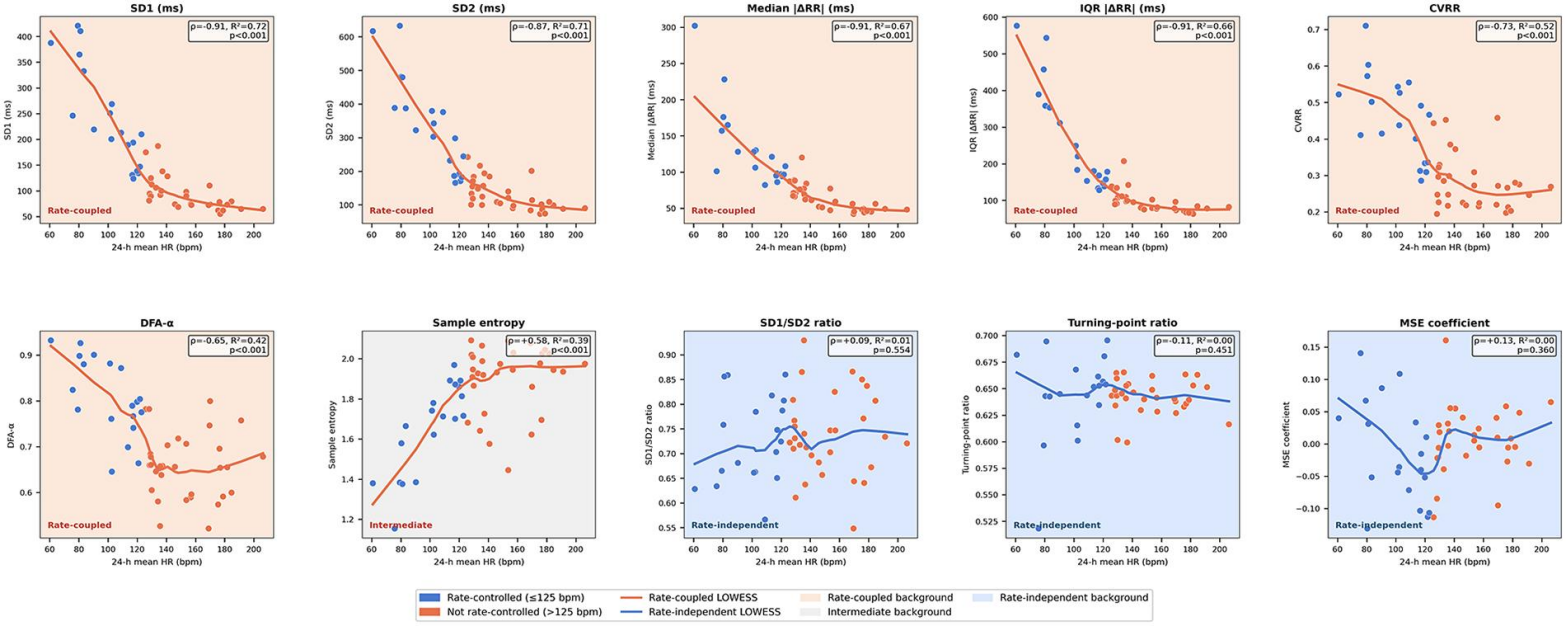
Scatterplots of each non-linear RR metric vs. 24-h meanHR (*n* = 50 dogs). Shaded backgrounds indicate metric class: orange = rate-coupled, blue = rate-independent. Blue points: rate-controlled dogs (meanHR ≤125 bpm); orange points: not rate-controlled. Locally weighted scatterplot smoothing (LOWESS) curves are shown. Spearman’s *ρ*, *R*^2^, and *p*-value are annotated. Rate-coupled metrics (top two rows) show strong monotonic relationships with meanHR; rate-independent metrics (bottom row, blue panels) show flat scatter. Note the positive *ρ* for SampEn—higher entropy at faster rates, an intermediate behavior. Turning-point ratio within-bin IQR ratio > 1.0 indicates variability that actually exceeds the global IQR within HR strata.

Spearman correlations with meanHR and within-bin IQR ratios are presented in Table 2. The metrics are partitioned into two classes (Figure 1). Rate-coupled metrics, including SD1 (*ρ* = −0.908), SD2 (*ρ* = −0.868), median|ΔRR| (*ρ* = −0.914), IQR|ΔRR| (*ρ* = −0.908), CVRR (*ρ* = −0.743), and DFA-*α* (*ρ* = −0.653), were all strongly correlated with meanHR (*R*^2^ = 0.42–0.72; all *p* < 0.001). Their within-bin IQR ratios were low (0.37–0.65), confirming that most of their variability was explained by the meanHR. SampEn was positively correlated with meanHR (*ρ* = +0.579, *R*^2^ = 0.39), indicating that higher rates were associated with higher apparent entropy, a mathematically predictable finding reflecting that at faster rates, absolute RR intervals are shorter and closer together, reducing the probability of template matches under fixed fractional tolerance.

**Table 2:** Rate dependence of non-linear RR metrics.

Metric	Spearman *ρ*	*p*-Value	*R*^2^ (metric∼meanHR)	Within-bin IQR ratio	Class
Median |ΔRR| (ms)	−0.914	<0.001	0.666	0.37	Rate-coupled
IQR |ΔRR| (ms)	−0.908	<0.001	0.665	0.57	Rate-coupled
SD1 (ms)	−0.908	<0.001	0.716	0.45	Rate-coupled
SD2 (ms)	−0.868	<0.001	0.708	0.45	Rate-coupled
CVRR	−0.743	<0.001	0.540	0.51	Rate-coupled
DFA-*α*	−0.653	<0.001	0.423	0.65	Rate-coupled
Sample entropy	+0.579	<0.001	0.392	0.64	Intermediate
SD1/SD2 ratio	+0.086	0.554	0.007	0.73	Rate-independent
Turning-point ratio	−0.109	0.451	0.003	1.16	Rate-independent
MScEn coefficient	+0.132	0.360	0.001	0.92	Rate-independent

Within-bin IQR ratio: average within-HR-quintile IQR divided by global IQR; ratio approaching 1.0 indicates that variability is independent of meanHR. Rate-coupled: *R*^2^ ≥ 0.25; rate-independent: *R*^2^ < 0.10.

Rate-independent metrics, including SD1/SD2 ratio (*ρ* = +0.086, *R*^2^ = 0.007), turning-point ratio (*ρ* = −0.109, *R*^2^ = 0.003), and MScEn coefficient (*ρ* = +0.132, *R*^2^ = 0.001), showed no significant correlation with meanHR (all *p* > 0.35). Their within-bin IQR ratios were high (0.73–1.16), indicating that most of their variability persists after controlling for mean rate.

Throughout, meanHR was computed as 60,000 ms ÷  mean RR interval, consistent with standard clinical Holter reporting. To assess whether results would differ materially using mean instantaneous HR (mean of 60,000/RRᵢ across individual beats), all Spearman correlations were repeated *post hoc* against mean instantaneous HR; the two HR measures were highly correlated (*ρ* = 0.976, *p* < 0.001), direction and rank-order of all metric-HR associations were preserved, and correlation magnitudes changed by |Δ*ρ*| ≤ 0.09 for all rate-coupled metrics (Supplementary Table S1). The largest change was observed for SampEn (|Δ*ρ*| = 0.12), consistent with partial mathematical confounding arising from the shared RR-interval basis of both quantities. Therefore, results are reported using meanHR throughout for consistency with standard clinical Holter reporting; the full comparison is provided in Supplementary Table S1.

### Jensen-gap index (*D*): validation and determinants

**Figure 2:**
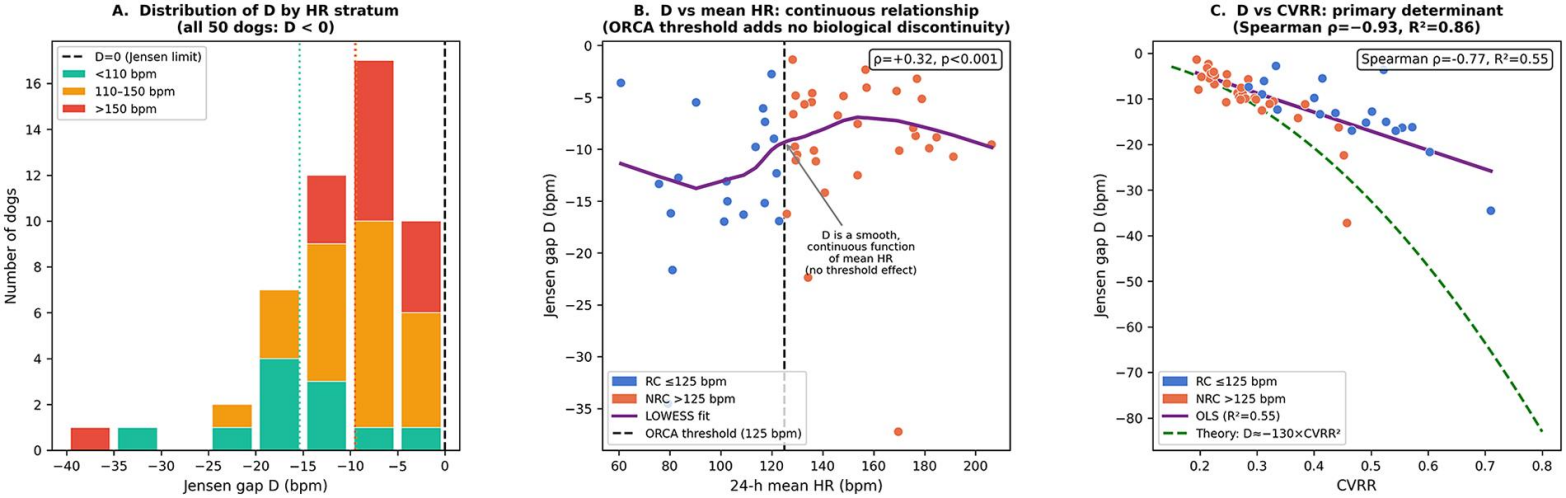
Jensen-gap index (*D*) across the cohort. **(A)** Distribution of *D* stratified by the meanHR stratum (<110, 110–150, >150 bpm). All values are negative (Jensen's inequality); the magnitude of *D* is largest at lower rates (wider RR dispersion). **(B)** *D* vs. continuous meanHR with LOWESS smoothing. The ORCA threshold (125 bpm, dashed line) shows no biological discontinuity—*D* is a smooth, monotonic function of HR. **(C)** *D* vs. CVRR. OLS regression (*R*^2^ = 0.863) and the theoretical approximation *D* ≈ −meanHR × CVRR^2^ (dashed) are shown, confirming CVRR as the primary determinant of *D*.

The Jensen-gap index (*D*) was negative in all 50 dogs (mean −13.4 bpm, median −11.2 bpm, SD 6.4 bpm, IQR [−16.7, −8.1], range −29.9 to −5.8 bpm; Shapiro–Wilk *p* = 0.0003, left-skewed). The overestimation, *E* = −*D*, averaged 13.4 bpm (median 11.2 bpm; range 5.8–29.9 bpm), equivalent to a mean relative overestimation of 11.6% (median 8.5%, maximum 43.9%). *D* was strongly predicted by CVRR (Spearman’s *ρ* = −0.934, *p* < 0.001; ordinary least squares (OLS) *R*^2^ = 0.863; slope = −46.3 bpm/unit CVRR), consistent with the theoretical approximation *D*/meanHR ≈ −CVRR^2^. *D* was also significantly stratified by HR stratum: mean *D* = −21.0 bpm in dogs with meanHR <110 bpm (*n* = 11), −12.0 bpm in those with meanHR 110–150 bpm (*n* = 24), and −10.0 bpm in those with meanHR >150 bpm (*n* = 15; Kruskal–Wallis *H* = 17.4, *p* = 0.0002; Figure 2). Importantly, *D* showed no discontinuity at the 125-bpm threshold. The relationship between *D* and continuous meanHR was smooth and monotonic (Spearman’s *ρ* = +0.544, *p* < 0.001), with no evidence of an inflection point at the ORCA threshold. After controlling for continuous meanHR, the partial Spearman correlation between *D* and rate-control status was −0.08 (*p* = 0.57).

### Limitations of the 125 bpm threshold as a rate-control surrogate

**Figure 3:**
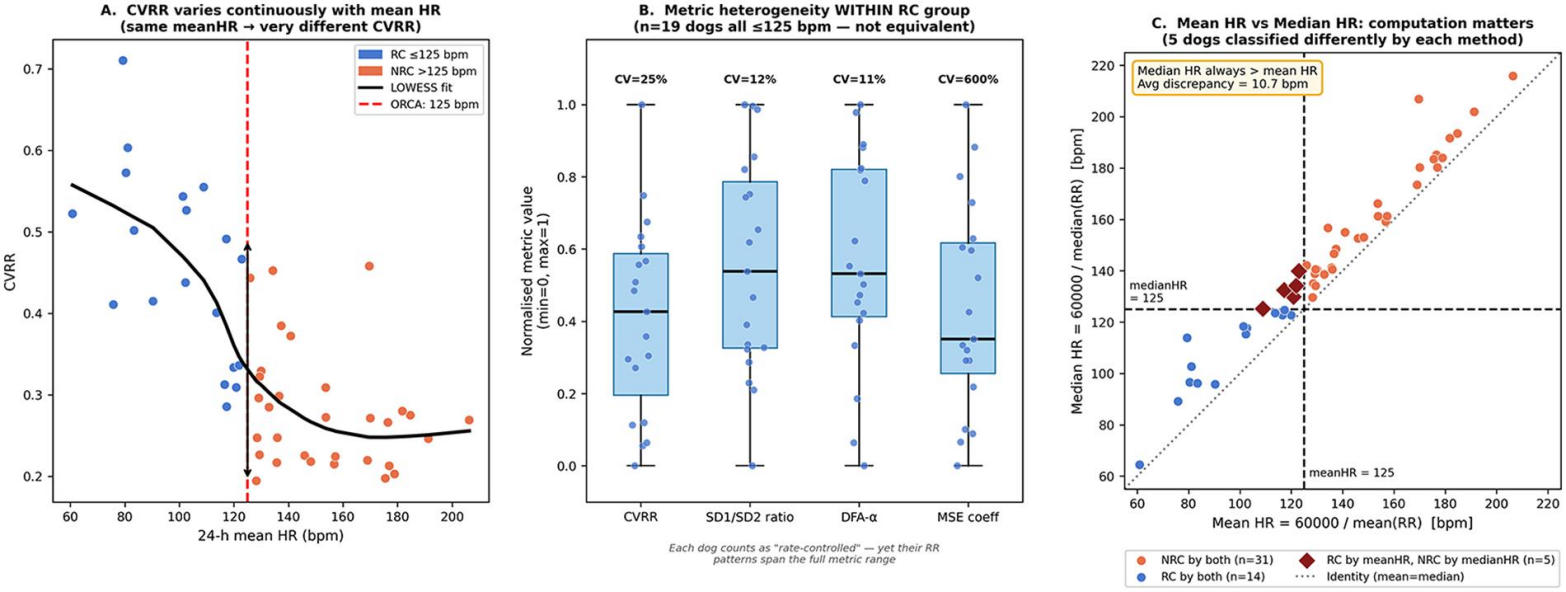
Limitations of the 125-bpm rate-control threshold. **(A)** CVRR vs. meanHR with LOWESS fit and ORCA threshold (red dashed line). CVRR varies continuously with meanHR; the threshold crosses a smooth gradient with no inflection. Dogs at the same meanHR can have widely different CVRRs. **(B)** Metric heterogeneity within the rate-controlled group (*n* = 19 dogs, all ≤125 bpm). Each metric is normalized to its population range (0–1); the coefficient of variation (CV%) is annotated. Despite the same rate-control label, dogs span nearly the full range of each irregularity metric. **(C)** MeanHR [60,000/mean(RR)] vs. medianHR [60,000/median(RR)] for all dogs. MedianHR is always higher than meanHR (median discrepancy 10.7 bpm). Red diamonds (*n* = 5) indicate dogs classified as rate-controlled by meanHR but uncontrolled by medianHR—a computationally ambiguous zone at the clinical threshold. Dashed lines: 125 bpm reference.

Five dogs (26% of rate-controlled dogs, 10% of the cohort) were classified as rate-controlled based on meanHR (≤125 bpm) but as uncontrolled based on medianHR (>125 bpm; Figure 3C). This misclassification arises because meanHR = 60,000/mean (RR) is lower than the medianHR by the left-skew of the RR distribution, reflecting the mathematical consequence of RR dispersion rather than a true rate control. If a Holter system reports mean instantaneous HR rather than HR from mean RR, the reported HR of the same dog would be higher by the Jensen gap (median 11.7 bpm near 125 bpm), potentially reclassifying a controlled dog as uncontrolled.

Among the 19 rate-controlled dogs (all with meanHR ≤125 bpm), heterogeneity in the RR irregularity structure was substantial and unresolved by the threshold: CVRR ranged from 0.285 to 0.748 [coefficient of variation (CV) 26%], SD1/SD2 ratio ranged from 0.566 to 0.860 (CV 12%), DFA-α ranged from 0.645 of 0.932 (CV 11%), and MScEn coefficient ranged from −0.131 to 0.140 (CV 600%). These metrics spanned nearly their full population ranges within this heart rate homogeneous group. Two dogs with identical meanHR values of approximately 102 bpm, for example, could differ in CVRR by >0.2, a difference corresponding to a Jensen gap discrepancy of approximately 8 bpm. The threshold is therefore collapsing biologically heterogeneous animals into a single clinical category.

### Rate-independent metrics and drug effects

Despite no significant difference in meanHR between predrug and postdrug dogs (128.5 vs. 134.3 bpm, *p* = 0.403), three rate-independent metrics differed significantly: the SD1/SD2 ratio was higher in postdrug dogs (0.747 vs. 0.710, *p* = 0.020), the MScEn coefficient was higher (0.024 vs. −0.018, *p* = 0.032), and DFA-α was lower (0.664 vs. 0.746, *p* = 0.047) (Table 3). As expected from its rate coupling, CVRR did not differ between groups (*p* = 0.403). Critically, the SD1/SD2 ratio and the MScEn coefficient did not differ between RC and NRC dogs (*p* = 0.780 and *p* = 0.246), confirming that they do not simply track with meanHR. These findings indicate that rate-control drugs reshape the RR irregularity structure independently of their effect on mean ventricular rate.

**Table 3:** Non-linear metric comparisons: predrug vs. postdrug and rate-controlled vs. not rate-controlled.

Metric	Predrug (*n* = 23) median [IQR]	Postdrug (*n* = 27) median [IQR]	*p*^†^	RC ≤125 (*n* = 19) median [IQR]	NRC >125 (*n* = 31) median [IQR]	*p*^†^
Rate-coupled metrics						
MeanHR (bpm)	128.5 [105.8–161.7]	134.3 [121.9–155.2]	0.403	102.6 [82.2–117.3]	148.2 [133.6–172.8]	<0.001*
CVRR	0.337 [0.258–0.496]	0.309 [0.235–0.425]	0.403	0.466 [0.384–0.544]	0.269 [0.226–0.319]	<0.001*
SD1 (ms)	112.0 [83.7–229.4]	118.3 [76.6–156.4]	0.533	212.9 [168.1–300.6]	89.4 [72.6–108.0]	<0.001*
SD2 (ms)	183.3 [116.1–359.2]	156.3 [98.7–204.8]	0.192	321.9 [211.2–387.7]	113.8 [97.6–155.3]	<0.001*
DFA-α	0.746 [0.658–0.814]	0.664 [0.598–0.768]	0.047*	0.798 [0.763–0.880]	0.657 [0.598–0.699]	<0.001*
Sample entropy	1.860 [1.680–1.981]	1.919 [1.698–1.971]	0.508	1.712 [1.482–1.837]	1.945 [1.863–2.022]	<0.001*
Rate-independent metrics						
SD1/SD2 ratio	0.710 [0.642–0.757]	0.747 [0.703–0.831]	0.020*	0.724 [0.662–0.797]	0.731 [0.689–0.789]	0.780
Turning-point ratio	0.643 [0.635–0.653]	0.645 [0.636–0.660]	0.668	0.652 [0.638–0.665]	0.643 [0.635–0.653]	0.280
MScEn coefficient	−0.018 [−0.048–0.007]	0.024 [−0.018–0.051]	0.032*	−0.036 [−0.062–0.036]	0.008 [−0.020–0.036]	0.246
Jensen gap						
Jensen gap *D* (bpm)	−11.2 [−18.5, −9.5]	−10.5 [−15.7, −7.9]	0.448	−15.9 [−22.6, −12.3]	−9.9 [−12.9, −7.5]	<0.001*
Partial rho *D*∼RC | meanHR	—	—	—	*ρ* = −0.08	*p* = 0.574	NS^‡^

* *p* < 0.05.

† Mann–Whitney *U*-test (two-sided).

‡ D∼RC difference is entirely explained by continuous meanHR; partial correlation after controlling for meanHR is non-significant. Predrug and postdrug groups constitute different dogs (cross-sectional); results are exploratory. Note that DFA-α is rate-coupled, and its significant RC/NRC difference is expected.

To illustrate the clinical relevance of rate-independent heterogeneity, Supplementary Figure presents 24-h Poincaré plots from two representative dogs with identical meanHR values but markedly different RR-interval cloud morphology. Dog 1 exhibited a larger, more diffuse cloud, whereas Dog 2 showed a more compact, elongated cloud. This example demonstrates that meanHR alone does not fully characterize the structure of RR-interval dynamics and that rate-independent metrics capture clinically meaningful heterogeneity that is not apparent to heart rate-based monitoring.

## Discussion

This study provides the first systematic characterization of the rate-dependence structure of non-linear RR interval metrics in a cohort of dogs with naturally occurring canine AF, with findings that translate directly to how rate-control monitoring is designed in both veterinary and human AF practice. Two interconnected findings emerge. First, most commonly reported non-linear RR metrics are mathematically coupled to mean ventricular rate and provide limited information beyond meanHR alone. Second, the 125-bpm rate-control threshold is computationally ambiguous and insufficient as a complete descriptor of the ventricular rate response during AF.

The strong coupling of absolute dispersion metrics (SD1, SD2, |ΔRR|) with meanHR reflects a physiological and mathematical constraint: as heart rate increases, the mean RR shortens, progressively narrowing the absolute range within which RR intervals can vary. This limits the additional clinical information these metrics provide beyond heart rate alone, accounting for most of the explained variance (*R*^2^ = 0.72–0.75). Reporting or comparing SD1 or SD2 across groups with different meanHR conveys limited insight into RR irregularity beyond that already captured by heart rate, a limitation equally applicable to Holter studies of human AF.

CVRR exhibited partial rate coupling (R^2^ = 0.54), reflecting partial correction for the absolute-scale constraint through normalization by the mean RR. CVRR quantifies the distributional asymmetry between the mean and median RR, a quantity formally related to the Jensen convexity gap. SampEn exhibited a positive correlation with meanHR (*ρ* =  +0.579, *R*^2^ = 0.39) under standard fixed fractional tolerance (*r* = 0.2 × SD). At faster ventricular rates, absolute RR intervals are shorter, reducing the absolute interinterval distance and thereby decreasing the probability of template matches under a tolerance scaled to the local standard deviation. This represents a recognized rate-dependence artifact of fixed fractional parameterization (7). This correlation warrants interpretive caution because SampEn is computed directly from the RR interval sequence from which meanHR is also derived, creating a shared mathematical basis that predisposes the two measures to associate independently of any biological mechanism. The SampEn finding should therefore be interpreted as hypothesis-generating pending validation using mean instantaneous HR as the comparator. The SD1/SD2 ratio, MScEn coefficient, and turning-point ratio demonstrated no significant rate coupling (*R*^2^ < 0.01; within-bin IQR ratios, 0.73–1.16). These metrics characterize the shape and temporal organization of RR irregularity, the ellipticity of the Poincaré cloud, the density of directional reversals in consecutive RR differences, and the scaling behavior of entropy across observation timescales. These properties are orthogonal to mean ventricular rate and are therefore sensitive to AV nodal conduction dynamics that is independent of rate. It should be noted that all metrics evaluated here are RR interval-based and characterize only the timing structure of the ventricular response. They carry no information about QRS or T-wave morphology, which represents a complementary dimension of ventricular response not captured by interval series alone.

The SD1/SD2 ratio was selected as the primary index of Poincaré plot ellipticity because it is scale-independent and comparable across a heterogeneous cohort. Qualitative morphological features of the Poincaré cloud, such as comet, torpedo, and fan patterns described in human AF, may encode additional structural information that is not fully captured by the SD1/SD2 ratio alone. As shown in Supplementary Figure S1, two dogs with virtually identical meanHR exhibited strikingly different cloud morphology, density, and non-linear metric profiles, underscoring that meanHR alone is an insufficient descriptor of ventricular response structure in AF. Integrating rate-independent metrics with conventional linear measures and visual Poincaré plot assessment may therefore provide a more complete characterization of AV nodal behavior in both canine and human AF.

Jensen's *D* was negative in all 50 dogs in this cohort, representing complete empirical confirmation of the mathematical prediction from Jensen's inequality applied to a strictly convex function of a non-constant random variable. The median discrepancy of 11.2 bpm, with individual values reaching 29.9 bpm, is not a biological finding; it is a computational artifact arising from the non-equivalence of 60,000/mean(RR) and mean(60,000/RRᵢ). The clinical implication is straightforward: a dog with a true mean RR-derived HR of 120 bpm may be reported as having a heart rate of 131 bpm by a Holter system that computes the mean instantaneous HR, resulting in erroneous classification as not rate-controlled. The partial correlation between *D* and rate-control status, after controlling for continuous meanHR, was *ρ* = −0.08 (*p* = 0.57), confirming that Jensen's *D* varies as a smooth, monotonic function of HR with no evidence of a biological discontinuity at the 125-bpm threshold. Holter platform developers and clinical investigators should specify the HR computation method implemented, as this choice can directly determine the classification of individual patients relative to any fixed HR threshold.

The ORCA study established that a lower 24-h meanHR is associated with longer survival in dogs managed with rate-control drugs, a clinically important observation. However, survival-based cutoffs derived from a single cohort reflect the statistical characteristics of that dataset rather than a confirmed biological discontinuity. Data-derived thresholds require independent prospective validation before being interpreted as physiological boundaries (11, 12), a principle equally applicable to rate-control targets in human AF trials. Three limitations of the 125-bpm threshold are as follows. First, all rate-coupled metrics varied smoothly and continuously with meanHR, with no detectable inflection near 125 bpm, consistent with a statistically derived rather than biologically grounded threshold. Second, the threshold collapses biologically heterogeneous dogs into a single group based on rate control status. For example, among the 19 rate-controlled dogs, CVRR ranged from 0.285 to 0.748, while rate-independent metrics spanned their full population range, indicating that dogs meeting the rate-control criterion were not physiologically equivalent. Third, the threshold is computationally non-reproducible: five dogs met the meanHR criterion (≤125 bpm) but not the medianHR criterion (≤125 bpm) because the two computations differ by the Jensen gap. A clinical threshold whose patient classification depends on unspecified software behavior cannot serve as a reproducible reference standard for veterinary or human clinical trials.

The present study does not claim that rate-independent metrics are superior to meanHR for predicting survival. Establishing such a determination will require prospective outcome data. Rather, our findings demonstrate that the 125-bpm threshold discards reproducibly, quantifiable information contained within the RR distribution that is statistically independent of meanHR. The more defensible interpretation of the ORCA findings is that lower meanHR is continuously associated with better survival, but dichotomization at any specific value imposes an arbitrary boundary on that continuous relationship. Whether the survival benefit is mediated solely by rate reduction, by drug-specific effects on RR irregularity structure, or by rate-independent AV nodal dynamics remains unresolved. Notably, the same interpretive limitation applies to human rate-control trials that use a single HR threshold as their primary endpoint.

### Rate-independent metrics and AV nodal drug effects

The finding that the SD1/SD2 ratio, DFA-α, and MScEn coefficient differ between predrug and postdrug dogs despite similar meanHR values in this study population is intriguing and supports the hypothesis that rate-control drugs may alter ventricular response irregularity beyond simple rate reduction, potentially through effects on AV nodal dynamics. Diltiazem and digoxin act through distinct electrophysiological mechanisms, which would be expected to produce distinct RR temporal patterns (13). The higher SD1/SD2 ratio and MScEn coefficient in treated dogs suggest a shift toward more complex multi-timescale irregularity, potentially reflecting richer modulation of AV nodal concealed conduction by drug-modified nodal electrophysiology. An important caveat is that diltiazem and digoxin also alter atrial electrophysiology and the pattern of impulse bombardment arriving at the AV node. The observed changes in the RR irregularity structure may therefore partially reflect alterations in the atrial forcing function rather than exclusively intrinsic AV nodal modifications. Computational modeling work in human AF has demonstrated that the AV nodal refractory period and conduction delay properties produce distinct RR irregularity signatures (14, 15). A prospective paired study with the same dog before and after drug initiation is needed to confirm drug-specific irregularity signatures.

### Translational context

Dogs with naturally occurring AF represent a well-validated translational model for human AF because the two species share comparable AV nodal anatomy, intact autonomic innervation, clinically relevant disease substrates, and susceptibility to tachycardia-mediated cardiomyopathy (16, 17). The framework distinguishing rate-coupled from rate-independent metrics established here has direct relevance to human AF research. Although standard heart rate variability metrics derived from RR intervals have been studied as outcome predictors in human AF, few studies have systematically identified metrics that are rate-coupled, a distinction critical when comparing groups with different mean rates or evaluating treatment effects. The rate-control debate in human AF mirrors the discussion in canine rate control. The RACE II trial demonstrated that lenient rate control (resting HR <110 bpm) was non-inferior to strict rate control (<80 bpm) with respect to a composite cardiovascular outcome; however, neither RACE II trial nor AFFIRM assessed whether the structure of RR irregularity, characterized by rate-independent metrics, differed between strategies or predicted outcomes independently of mean rate (18, 19). The present finding that rate-independent metrics (SD1/SD2 ratio, MScEn) differed between drug-naive and drug-treated dogs despite similar meanHR values raises the hypothesis that AV nodal-targeted drugs produce distinct signatures of RR irregularities. Prospective evaluation in human AF cohorts is warranted.

The Jensen-gap finding is not species-specific: any Holter system that reports mean instantaneous HR will systematically overestimate HR relative to 60,000/mean(RR) in proportion to RR dispersion. In this cohort, the discrepancy reached 29.9 bpm; the patients with the most irregular ventricular responses are most affected, precisely the population in which rate-control classification matters most. As ambulatory monitoring endpoints become standard in human AF trials, the specification of the HR computation method is needed to enable comparison of results across studies.

### Limitations

This study has several important limitations. Its retrospective, cross-sectional design precludes causal inference; the predrug and postdrug groups comprised different dogs and may have differred with respect to disease severity, drug selection bias, timing of Holter application, and breed composition. With *n* = 50 total dogs and subgroup sizes as small as *n* = 9, all clinical subgroup analyses are underpowered and should be treated as hypothesis-generating. The VPC-inclusive computation of all metrics may introduce noise for dogs with high ectopic burden (18/50 dogs with V% > 5). However, an initial exploratory comparison of non-linear RR metrics, with and without ventricular origin beats included, showed no significant differences in the quantified metrics. The SampEn tolerance *r* = 0.2 × SD may not adequately adjust for rate-dependent changes in the RR scale, contributing to the apparent positive *ρ* between SampEn and meanHR. Critically, this cohort was managed according to routine clinical practice and was not prospectively titrated toward a 125-bpm target; therefore, the retrospective classification of dogs as rate-controlled (meanHR ≤125 bpm) reflects an observed state rather than the outcome of deliberate rate management and cannot be interpreted as equivalent to the prospective rate-control arms of dedicated clinical trials.

Non-linear RR interval metrics, particularly entropy-based measures (and DFA-α), are sensitive to ECG artifacts, including oversensed beats that generate spuriously short RR intervals or missed beats that generate anomalous long intervals. Such artifacts that introduce non-physiological sequential patterns can artificially alter regularity estimates. In this study, all recordings were reviewed by a board-certified veterinary cardiologist with beat-by-beat annotation, providing a high level of quality control. Nevertheless, artifact sensitivity represents a critical practical consideration for clinical implementation of these metrics in automated or large-scale Holter analysis. An additional limitation is that the cardiac substrate was not formally evaluated as a modifier of non-linear RR metrics. Dogs with dilated cardiomyopathy, myxomatous mitral valve disease, and lone AF may differ in autonomic tone, AV nodal refractory period, and atrial impulse bombardment pattern, each of which could independently shape the structure of ventricular RR irregularity. Future studies with larger, more uniform disease cohorts are needed to assess whether rate-independent metrics are substrate-dependent.

## Conclusions

In dogs with naturally occurring AF, non-linear RR metrics can be partitioned into rate-coupled metrics, which are mathematically constrained by mean ventricular rate, and rate-independent metrics, which characterize irregularity structure orthogonally to meanHR. The Jensen-gap index (*D*) was universally negative across all dogs, a finding consistent with mathematical theory, and varied as a smooth, continuous function of meanHR with no evidence of biological discontinuity at the ORCA threshold of 125 bpm. The 125-bpm threshold is computationally ambiguous, collapses heterogeneous RR distributions into a single category, and imposes an arbitrary dichotomy on a continuous relationship. Rate-independent metrics (SD1/SD2 ratio, MScEn coefficient) differ between drug-naive and treated dogs despite similar mean rates, suggesting that they capture aspects of RR irregularity structure beyond rate reduction. Future prospective studies of canine AF should include both meanHR and rate-independent irregularity metrics, specify the exact computational method used to derive HR, and test whether irregularity metrics predict clinical outcomes, including within rate-controlled dogs, independently of the binary rate-control threshold. Confirmation of drug-specific RR irregularity signatures will require a prospective within-dog paired design, with Holter evaluation performed before and after initiation of rate-control drug therapy in the same patient.

## Data Availability

The raw data supporting the conclusions of this article will be made available by the authors, without undue reservation.

## References

[1] Menaut P Bélanger MC Beauchamp G Ponzio NM Moïse NS. Atrial fibrillation in dogs with and without structural or functional cardiac disease: a retrospective study of 109 cases. J Vet Cardiol. (2005) 7(2):75–83. 10.1016/j.jvc.2005.07.002 19083323

[2] Moe GK Abildskov JA. Atrial fibrillation as a self-sustaining arrhythmia independent of focal discharge. Am Heart J. (1959) 58(1):59–70. 10.1016/0002-8703(59)90274-1 13661062

[3] Pedro B Mavropoulou A Oyama MA Linney C Neves J Dukes-McEwan J. Optimal rate control in dogs with atrial fibrillation—ORCA study—multicenter prospective observational study: prognostic impact and predictors of rate control. J Vet Intern Med. (2023) 37(3):887–99. 10.1111/jvim.16666 37128174 PMC10229328

[4] Pedro B Mavropoulou A Oyama MA Linney C Neves J Dukes-McEwan J. Longitudinal analysis of echocardiographic and cardiac biomarker variables in dogs with atrial fibrillation: the ORCA II study. J Vet Intern Med. (2024) 38(4):2076–88. 10.1111/jvim.17120 38877661 PMC11256134

[5] Jensen JLWV. Sur les fonctions convexes et les inégalités entre les valeurs moyennes. Acta Math. (1906) 30:175–93. 10.1007/BF02418571

[6] Brennan M Palaniswami M Kamen P. Do existing measures of Poincaré plot geometry reflect nonlinear features of heart rate variability? IEEE Trans Biomed Eng. (2001) 48(11):1342–7. 10.1109/10.959330 11686633

[7] Richman JS Moorman JR. Physiological time-series analysis using approximate entropy and sample entropy. Am J Physiol Heart Circ Physiol. (2000) 278(6):H2039–49. 10.1152/ajpheart.2000.278.6.H2039 10843903

[8] Costa M Goldberger AL Peng C-K. Multiscale entropy analysis of complex physiologic time series. Phys Rev Lett. (2002) 89(6):068102. 10.1103/PhysRevLett.89.068102 12190613

[9] Costa M Goldberger AL Peng C-K. Multiscale entropy analysis of biological signals. Phys Rev E. (2005) 71(2 Pt 1):021906. 10.1103/PhysRevE.71.021906 15783351

[10] Peng C-K Havlin S Stanley HE Goldberger AL. Quantification of scaling exponents and crossover phenomena in nonstationary heartbeat time series. Chaos. (1995) 5(1):82–7. 10.1063/1.166141 11538314

[11] Altman DG Lausen B Sauerbrei W Schumacher M. Dangers of using “optimal” cutpoints in the evaluation of prognostic factors. J Natl Cancer Inst. (1994) 86(11):829–35. 10.1093/jnci/86.11.829 8182763

[12] Lausen B Schumacher M. Maximally selected rank statistics. Biometrics. (1992) 48:73–85. 10.2307/2532740

[13] Fujiki A Mizumaki K Tani M. Effects of diltiazem on concealed atrioventricular nodal conduction in relation to ventricular response during atrial fibrillation in anesthetized dogs. Am Heart J. (1993) 125(5 Pt 1):1284–9. 10.1016/0002-8703(93)90996-M 8480579

[14] Karlsson M Platonov PG Ulimoen SR Sandberg F Wallman M. Model-based estimation of AV-nodal refractory period and conduction delay trends from ECG. Front Physiol. (2024) 14:1287365. 10.3389/fphys.2023.1287365 38283279 PMC10811553

[15] Sandberg F Corino VDA Mainardi LT Ulimoen SR Enger S Tveit A. Non-invasive assessment of the effect of beta blockers and calcium channel blockers on the AV node during permanent atrial fibrillation. J Electrocardiol. (2015) 48(6):861–6. 10.1016/j.jelectrocard.2015.07.019 26275982

[16] Sugumar H Prabhu S Voskoboinik A Kistler PM. Arrhythmia induced cardiomyopathy. J Arrhythm. (2018) 34(4):376–83. 10.1002/joa3.12094 30167008 PMC6111481

[17] Nattel S Burstein B Dobrev D. Atrial remodeling and atrial fibrillation: mechanisms and implications. Circ Arrhythm Electrophysiol. (2008) 1(1):62–73. 10.1161/CIRCEP.107.754564 19808395

[18] Clark DM Plumb VJ Epstein AE Kay GN. Hemodynamic effects of an irregular sequence of ventricular cycle lengths during atrial fibrillation. J Am Coll Cardiol. (1997) 30(4):1039–45. 10.1016/S0735-1097(97)00254-4 9316536

[19] Daoud EG Weiss R Bahu M Knight BP Bogun F Goyal R. Effect of an irregular ventricular rhythm on cardiac output. Am J Cardiol. (1996) 78(12):1433–6. 10.1016/S0002-9149(97)89297-1 8970422

