# Supplementary material for: Non-linear RR interval metrics in canine atrial fibrillation and their relationship to 24-h mean heart rate
Source: Front Cardiovasc Med. 2026 Jul 16;13:1890025. doi: 10.3389/fcvm.2026.1890025 (PMC13422497; doi:10.3389/fcvm.2026.1890025)
Supplement: Supplementary file 1 [file Datasheet1.pdf]

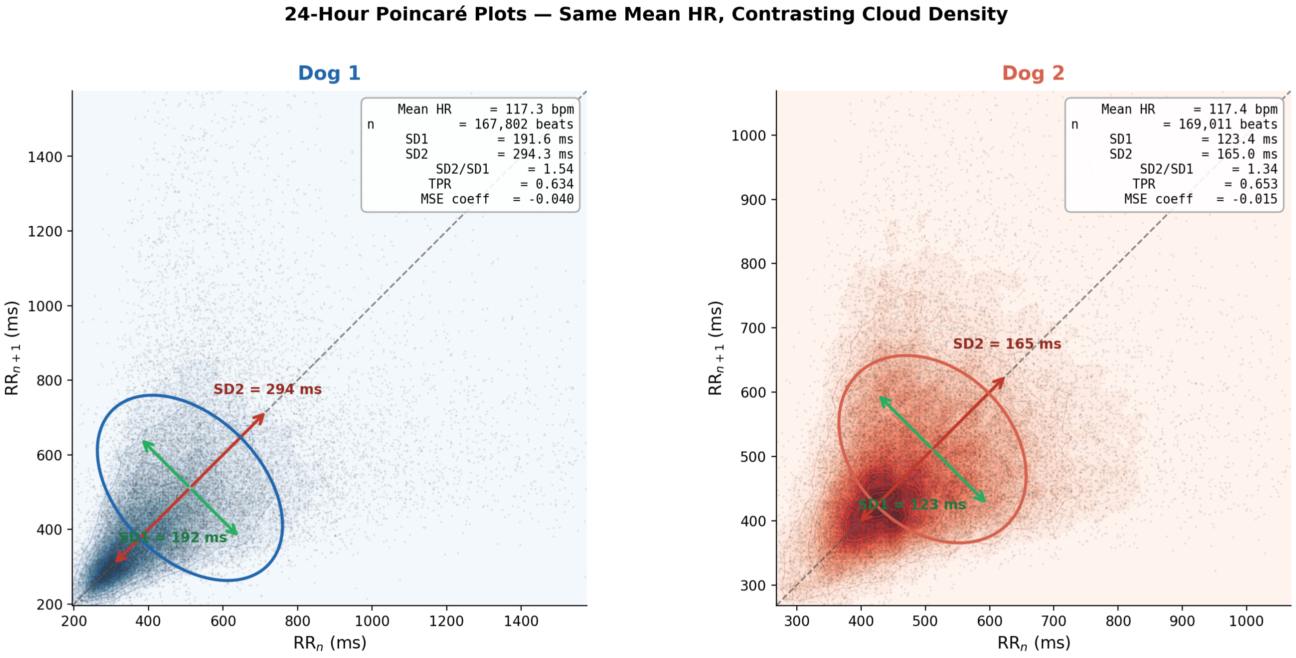

Supplementary Figure 1.

Twenty-four-hour Poincaré plots from two dogs with identical mean heart rate but contrasting RR-interval cloud density. Successive RR-interval pairs (RR_n vs RR_{n+1}, ms) are plotted for the full 24-hour Holter recording. Colour intensity reflects kernel density. The fitted ellipse (coloured outline) illustrates SD1 (green arrows; short-term beat-to-beat variability, perpendicular to the line of identity) and SD2 (red arrows; long-term variability, along the line of identity). The dashed diagonal is the line of identity. Despite virtually identical mean heart rates (117.3 vs 117.4 bpm), Dog 1 showed a markedly larger, more diffuse cloud (SD1 192 ms, SD2 294 ms, SD2/SD1 1.54) compared with the compact cloud of Dog 2 (SD1 124 ms, SD2 165 ms, SD2/SD1 1.34). Dog 1 also had a lower turning point ratio (0.634 vs 0.653) and more negative multiscale entropy coefficient (–0.040 vs –0.015), indicating reduced rhythmic complexity. These findings demonstrate that mean heart rate alone does not characterize the full structure of RR-interval dynamics, and that rate-independent metrics capture clinically meaningful heterogeneity invisible to heart rate-based monitoring.
